# Facial Reconstruction Using Facial Artery Myomucosal Flap: A Comprehensive Review

**DOI:** 10.7759/cureus.42060

**Published:** 2023-07-18

**Authors:** Bader Fatani, Abdulrahman I Alhilal, Hadeel H Alzahrani, Raghad R Alkhattabi, Mariam Alhindi

**Affiliations:** 1 Dentistry, College of Dentistry, King Saud University, Riyadh, SAU; 2 College of Dentistry, Princess Nourah Bint Abdulrahman University, Riyadh, SAU; 3 Oral and Maxillofacial Surgery, King Saud University, Riyadh, SAU

**Keywords:** facial artery flap, facial artery myomucosal, reconstruction, facial defects, famm flap

## Abstract

The facial artery myomucosal (FAMM) flap is a type of facial flap that is constructed with the buccal mucosa and submucosa along with a portion of the buccinator muscle, which is connected to nearby blood vessels to maintain its blood supply. It is a versatile and reliable option for head and neck reconstruction, particularly in oral cavity defects. This flap is employed in the reconstruction of the floor of the mouth, alveolar cleft surgery, and tongue defect repair. Previous studies have discussed the use of FAMM flaps for facial reconstructions. However, there are no current and updated comprehensive reviews discussing the use of FAMM flaps in facial reconstruction. In light of this, this study aimed to review all relevant studies that discuss the use of FAMM flaps in facial reconstruction.

## Introduction and background

Reconstructing deformities in the head and neck is a difficult task since the structures of these areas are both extremely apparent and very functional [1]. In order to restore shape and function following head and neck injuries and defects, complicated abnormalities must be repaired [2]. Various reconstructive procedures, including skin grafts, pedicled flaps, and free flaps, can be performed based on the nature and extent of the deformities [2]. It can be time-consuming and requires surgical skills in microsurgery to harvest free flaps [1,2]. The most common and complex head and neck abnormalities may still be repaired using free flaps [1]. Pedicled flaps can be used to successfully restore small to medium-sized head and neck deformities with little repercussions on the donor site [1,2].

In recent years, the use of regional pedicled flaps, such as the submental flap, supraclavicular flap, or facial artery musculomucosal (FAMM) flap, has been more often reported in the literature [2]. FAMM flap refers to an axial composite flap based on the facial artery in the buccal area [3]. The primary goal as per the first description by Pribaz et al. was to rebuild the lip and vermillion by using a flap that was superiorly based on retrograde flow from the facial artery [3]. The benefits of this pedicled intraoral flap vastly exceed its drawbacks [2]. This flap has several advantages such as dependability, the lack of an externally visible scar, and little donor site morbidity [4]. However, it has certain drawbacks, such as the potential for scar contracture at the donor site, its small size, and the requirement for a second-stage treatment in certain patients to segment the pedicle [4]. The range of applications for this flap has lately undergone several alterations to enhance its size and length or eliminate a two-stage operation [2]. The mandibular periosteum or masseteric fascia can be added to the design to lengthen the conventional FAMM flap and provide access to more posterior skull base regions [4]. A mucosal defect can be repaired with mucosa using the FAMM flap [1]. While previous clinical studies have discussed the use of FAMM flaps for facial reconstructions, there is a paucity of current and updated comprehensive reviews discussing the use of FAMM flaps in facial reconstruction. Hence, this study aimed to review the use of FAMM flaps in facial reconstruction.

## Review

Methodology

This review article involved an assessment of published studies discussing the use of FAMM flaps for head and neck reconstruction. Several databases such as PubMed, Web of Science, and Google Scholar were used to gather the most relevant papers on this topic. The search employed a range of keywords such as facial artery myomucosal flaps, musculomucosal flap, and head and neck reconstruction. By using this method, all the studies discussing the use of FAMM flaps for head and neck reconstruction were obtained. We included all the relevant studies discussing FAMM flaps and their indication, contraindication, treatment planning, surgical technique, survival rate, and complications in the inclusion criteria. The studies that had poor methodological quality and those with outdated and insufficient data were excluded. The initial screening elicited 167 studies. After applying our inclusion criteria, the most relevant articles were selected and used in the current review. This review ultimately involved 52 papers related to the use of FAMM flaps for head and neck reconstruction.

Head and neck reconstruction 

For small to medium-sized abnormalities, mainly in the head, neck, and oral cavity, FAMM flaps have been utilized extensively [2]. They have a variety of clinical uses. In addition to repairing cleft palates, septal perforations, and osteoradionecrosis, they are frequently used for reconstructing defects left behind following tumor ablation [2]. The palatal island flap and FAMM flap are two local choices for repair that have a low risk of donor-site complications for mild mucosal defects [1]. The oral cavity, the septum, the palate, and the throat may all be repaired with the FAMM flap when it is positioned superiorly (distally near the continuation of the angular artery) or inferiorly (proximally near the jaw) [3]. Treatment for individuals with severe oropharyngeal and nasopharyngeal stenosis, such as full stenosis, can be accomplished with the FAMM flap as an efficient reconstruction tool [3].

In the reconstruction of the skull base, the superiorly pedicled FAMM flap is a practical choice [4]. The frontal sinuses, the sella turcica, the fovea ethmoidalis, and the planum sphenoidale can all be reached with the FAMM flap [4]. The vermillion and tonsillar fossa, alveolus, the floor of the mouth, lower lip, and other abnormalities can all be treated with the inferiorly based FAMM flap [5]. Recently, there has been a surge in the usage of this flap to treat post-ablation maxillofacial abnormalities. An inferiorly based FAMM flap be employed only if the facial artery and, if feasible, the linguofacial vein have been preserved in neck dissections [5]. The palate, upper vestibule, nasal cavity, and orbit have all been effectively reconstructed using the superiorly based FAMM flap [6].

Facial artery myomucosal flap 

The FAMM flap was first introduced by Pribaz and colleagues in 1992, and since then, it has been utilized for repairing various types of intraoral defects [7,8]. The FAMM island flap (FAMMIF) is a type of flap made up of the buccal mucosa and submucosa along with a portion of the buccinator muscle, which is connected to nearby blood vessels to maintain its blood supply [9]. An axial pattern pedicled flap uses tissue that is ensured to have a blood supply by incorporating an artery in both the base and length of the flap [10]. The FAMM flap is typically employed to repair defects in various parts of the mouth and throat, including the floor of the mouth, palate, alveolar ridge, lip, and oropharynx [11, 12]. Previous studies have indicated that the FAMM flap offers several benefits as a reconstructive option: it avoids any visible scarring from the harvesting process; it has a good range of movement and can be used to reconstruct multiple areas; it is flexible and thin; it provides functional mucosal tissue; it is effective in repairing damage in previously radiated patients; and it has a robust blood supply that can withstand postoperative radiotherapy [13]. The flap can be based either inferiorly on the facial artery or superiorly on the retrograde flow of the angular artery [13]. The FAMM flap that is based superiorly is crucial for reconstructing specific defects that affect the oral cavity and other remote areas of the head and neck region [14]. Additionally, venous drainage of the FAMM flap is not exclusively through the facial vein, but often through the submucosal plexus [13].

Pan et al. investigated the role of the FAMM flap in reconstructing floor-of-mouth and tongue defects [15]. The authors described that the FAMM flap offers several benefits for repairing defects in the tongue and floor of the mouth, including a straightforward operation, effective repair, a high survival rate of the flap, and minimal injury at the donor site [15]. For the closure of a wide palatal fistula that occurs repeatedly, the FAMM flap is a dependable and adaptable option that offers comparable tissue [16]. The FAMM flap is a reliable and versatile flap that is used to reconstruct damaged tissue and is proven to be an effective option for addressing recurrent fistulas in the hard palate. [17]. Ashtiani et al. have indicated that the FAMM flap is a dependable and beneficial technique that can be utilized as a suitable substitute for treating extensive, scarred, and recurring palatal fistulas [18].

Careful treatment planning is essential in these cases [19]. Benjamin et al. concluded that the FAMM flap is a dependable choice for fixing small, oral tongue defects without causing functional issues [20]. It is also a great option for repairing medium-sized defects, where other methods may result in tissue tethering and functional impairment [20]. It can also be a good choice for larger, medium-sized defects where free tissue transfer may be excessive [20]. The study by Lee et al. suggests that the spacer FAMM flap is a new version of a well-known method that can repair oronasal fistulas in one go, and it can be utilized on both sides. Additionally, the flap extends the palate length by pushing back the velum with a pedicle inset, which could have an effect on velopharyngeal function [21]. Moreover, a superiorly pedicled FAMM flap can be a viable choice for reconstructing the skull base [4]. Mannino et al. showed that the FAMM flap is effective in repairing nasopharyngeal stenosis and velopharyngeal insufficiency caused by prior surgery or scarring [3]. Although it has many benefits, the widespread utilization of the FAMM flap has been restricted due to insufficient knowledge and challenges in consistently raising a reliable flap safely [22]. A retrospective study by Massarelli et al. concluded that the buccinator myomucosal island flaps are a useful and efficient choice for reconstructive surgery in the oropharynx, with a short operation time and a low rate of complications at the donor site [23]. Furthermore, Rahpeyma et al. suggested that although the morbidity rate at the donor site for buccinator-based myomucosal flaps is relatively low, around 20% of patients experienced a slight decrease in mouth opening [24].

According to Rahpeyma et al., a thin, elongated buccal myomucosal flap that excludes the facial artery and vein has the potential to survive [25]. Massarelli et al. concluded that the folded tunnelized FAMMIF is a simple and effective technique for reconstructing the soft palate after surgery. It can be performed through the mouth and has minimal negative effects on the donor site while achieving the two main objectives of soft palate reconstruction [26]. According to Woo et al., using the buccinator myomucosal flap is an excellent choice for repairing moderate-sized oral cavity defects due to its versatility, short operating time, option for using tissue that includes mucosa, and low donor site complications. Additionally, the accompanying node dissection of level Ib should not be considered a contraindication to this flap, as it receives a blood supply from the buccal artery [27]. Saad et al. observed a favorable outcome seven months after utilizing the FAMM flap as a reconstructive option for socket contracture, resulting in a relatively uncomplicated procedure, which indicates a new usage for the FAMM flap in this aspect [28]. The FAMM flap has a high survival rate and offers advantages such as shorter operating time, fewer injuries to the patient, and greater patient acceptance, particularly among older patients, when compared to free flap surgery. In cases of intraoral repair, it is superior to the submental myocutaneous flap, as the tissue of the FAMM flap is similar to other oral tissues [29].

According to Joseph et al., the islanded FAMM flap offers a less complex and cost-effective option with acceptable donor site risks for reconstructing lateral oral tongue defects compared to fasciocutaneous free flaps, and it also provides good aesthetic outcomes [30]. Island flaps based on the facial artery can effectively reconstruct defects in the head and neck area, with minimal harm to the donor site. These flaps provide good aesthetic outcomes, are easy to harvest, and have a satisfactory amount of bulk and mucosa. Not only is reconstruction done in one stage, but it is also a fast, cost-effective, and viable option for oral cavity, oropharynx, larynx, hypopharynx, and tracheal mucosal defects [31]. The facial artery-based island flap can be used for small reconstructions in the upper hypopharynx. This method is advantageous due to its accessibility, low harm to the donor site, and similarity to recipient tissue [32]. Rahpeyma et al. showed that a superiorly based FAMM flap is a good option when the fistula in the palate is continuous with the alveolar cleft [33]. Furthermore, they explained that a musculomucosal flap of the facial artery that is only supported by the skeletonized artery alone, known as an island FAMM flap, is not considered viable for use in clinical settings due to biological factors [34]. The inferiorly based buccinator myomucosal island flap was first described by Zhao in 2003. This flap is designed as an axial-pattern flap and consists of certain fibers from the orbicularis oris muscle and a portion of the buccinator muscle, which is then covered by the buccal mucosa [35].

Indications and contraindications

The FAMM flap is a versatile and reliable option for head and neck reconstruction, particularly in oral cavity defects. Indications for the use of the FAMM flap include reconstruction of the floor of the mouth [8], alveolar cleft surgery [33], nasopharyngeal reconstruction [32], and tongue defect repair [29]. In addition, the FAMM flap has been applied in lower eyelid fornix deepening [28] and closure of large anterior palatal fistulas [36]. Contraindications for using the FAMM flap include a compromised or absent facial artery, as the flap relies on the blood supply provided by the facial artery [34]. Additionally, patients with a history of radiation therapy in the area may have compromised vasculature, which can pose a risk to flap survival [37]. Oncologic safety is another consideration, as the FAMM flap should not be used in cases where there is a risk of transferring malignant cells from the donor site to the recipient site [12].

Patient selection and treatment planning 

Several studies have confirmed the utility of the FAMM flap in the reconstruction of intraoral and neck regions defects of various sizes. It is mostly used to reconstruct defects resulting from tumor removal, and other indications including cleft palate/lip repair [2]. This flap has been successfully utilized by recent studies in reconstructing other head and neck regions, such as lower eyelid reconstruction [14,28]. The FAMM flap can also be used to manage simple oral trauma such as labial mucosa loss after dentoalveolar fracture [25]. Additionally, it can be used to manage complex head and neck trauma such as gunshots [38], or motor vehicle accidents (MVA) [39].

Once the patient is selected and the FAMM flap is decided to be the best option for the case, preoperative preparation is important to optimize the results [19,40]. Preoperative preparation consists of the localization of the facial artery using Doppler ultrasound [22,41,42], and localizing the parotid duct (Stensen’s duct) [17]. Table 1 summarizes the patient data from the case reports included in this review, in which the FAMM flap was used to reconstruct oral, nasal, oropharyngeal, and eyelid reconstruction, along with the cause of the defect, the indication for the use of the FAMM flap, and the complications that occurred.

**Table 1:** Summary of case reports in which FAMM ﬂaps were used for reconstruction FAMM: facial artery musculomucosal

Article	Patient	Cause of defect	Indication	FAMM flap	Complications
Shivanand et al., 2018 [8]	-	Mucoepidermoid carcinoma of right submandibular and sublingual glands	Reconstruction of the floor of the mouth and ventral tongue defect	Raised from the left buccal mucosa and tunneled through the mylohyoid muscle into the floor of the mouth	-
Frisch, 2017 [32]	58-year-old male	Laryngopharyngectomy of squamous cell carcinoma (SCC) in the right aryepiglottic fold	Reconstruction of a hypopharynx defect	Raised from the right cheek and tunneled it lateral to the mandibular bone	Minor fistula unrelated to the flap
Saad et al., 2021 [28]	28-year-old male	Eye enucleation of retinoblastoma	Reconstruction of the lower eyelid	Raised from the right buccal mucosa and tunneled to the right lower eyelid	Trismus
Massarelli et al., 2013 [42]	76-year-old male	Squamous cell carcinoma (SCC) of the left cheek	Reconstruction of left cheek mucosa	Harvested from the right cheek as a free flap and rotated to the left cheek through a paramandibular tunnel in the soft cheek tissue	Trismus
Jeong et al., 2017 [37]	59-year-old female	Flap necrosis after fistula closure using two-flap palatoplasty	Reconstruction of the secondary soft palatal defect	Harvested and tunneled submandibularly on the lingual side of the mandible	Trismus
Massarelli et al., 2013 [26]	67-year-old male	Squamous cell carcinoma (SCC) of the uvula	Reconstruction of a soft palate defect	Harvested the flap from the right cheek and rotated through a tunnel in the right anterior pillar of the tonsillar fossa	Trismus
Massarelli et al., 2017 [38]	58-year-old male	Self-inflicted gunshot injury of the left cheek	Reconstruction of a full-thickness nasolabial defect	Harvested from the right cheek and transported to the contralateral side	-
Rahpeyma et al., 2016 [25]	42-year-old female	Anterior mandibular dentoalveolar fracture	Replacing the lost labial mucosa	Inferiorly based partial thickness FAMM	-
Pompei et al., 2016 [43]	50-year-old male	Squamous cell carcinoma (SCC) carcinoma of the soft palate	Reconstruction of an extensive palatal defect	Bilateral transverse (t)-FAMM flap was transposed superoposteriorly and sutured to the residual mucosa of the hard palate	-
Xie et al., 2016 [6]	71-year-old male	Large parapharyngeal and clival chordoma	Reconstruction of nasopharyngeal and skull base defect	Harvested an extended superiorly based FAMM ﬂap transferred through a Caldwell-Luc fenestration, through the maxillary sinus to the nasopharynx	-
Wang et al., 2018 [2]	61-year-old male	Squamous cell carcinoma (SCC) in the left nose region	Reconstruction of the upper lip	Harvested from the right intraoral cheek, the buccinator of the FAMM flap was sutured to the upper orbicularis muscles	-
Rahpeyma and Khajehahmadi, 2015 [39]	17-year-old male	Motor vehicle accident (MVA)	Reconstruction of a large full-thickness nasal defect	Harvested a superiorly based buccinator myomucosal from the ipsilateral buccal region to the nose through the nasal floor	Minor nostril stenosis
Ishak et al., 2018 [16]	10-year-old male	Recurrent oronasal fistula after bilateral cleft lip and palate repair	Reconstruction of a recurrent oronasal fistula	Advancement of the right FAMM flap	-
Akali et al., 2020 [13]	32-year-old male	Laryngectomy of a chondrosarcoma	Reconstruction of a partial pharyngeal defect	An islanded FAMM harvested from the left cheek tunneled down into the neck	Mild marginal mandibular nerve paresis

Surgical approach 

The FAMM flap technique enables the reconstruction of damaged oral tissues using minimal donor tissue, without the problems associated with other types of flaps, such as excessive bulk, scarring, and long recovery times. The anatomy of the flap is well-established, and its use does not interfere with thorough lymph node examinations. Experienced reconstructive surgeons can easily learn and utilize this method, and it is recommended for all practitioners in the field [43,44]. The FAMM flap technique requires less preoperative preparation compared to free tissue transfer. The most crucial diagnostic step is to check the Doppler signal on the facial artery to ensure its functionality, particularly in patients who have undergone neck surgery or radiation therapy.

The conventional approach for FAMM flap harvesting involves identifying the facial artery, which can be accomplished using either of the two methods. The first method involves the distal identification of the facial artery; the distal part of the flap is cut through the mucosa, submucosa, and buccinator muscle. Once the facial artery is located, it is clipped and sectioned distally. The second method involves the anterior identification of the facial artery; an incision is made 1 cm behind the oral commissure, going through the mucosa, submucosa, and orbicularis oris muscle to locate the superior labial artery. The superior labial artery is then traced backward, in a retrograde manner, until it reaches the facial artery.

During careful dissection, the "Y-shaped" junction of the three vessels (facial, superior labial, and nasal lateral arteries) is often observed, unless there are variations in the course of the facial artery. Ligation of the superior labial artery should only be performed when the angular artery is identified [2]. The flap is gathered beneath the facial artery by including the buccinator muscle and part of the orbicularis oris near the oral commissure. The facial artery must remain connected to the surrounding tissues throughout its length. Collateral vessels are clipped and divided as the dissection progresses from distal to proximal. Venous drainage relies on a submucosal plexus, and hence including the facial vein is not necessary. However, if the facial vein is not included, a 2-cm soft tissue base should be maintained for adequate venous drainage. Most of the harvesting steps for the inferiorly based FAMM flap also apply to the superiorly based FAMM flap. The base of the superiorly based FAMM flap is located near the superior gingival labial sulcus close to the alar margin, while the distal portion of the flap lies at the level of the retromolar trigone inferiorly. If the superior labial artery, which is a branch of the facial artery, is sufficient for retrograde flow, the FAMM flap can be based on it instead of the angular artery. If the flap is less than 3 cm wide, it can be closed primarily. If there is a concern about potential contracture due to excessive tension, the donor site can be skin grafted, left to granulate, or closed with buccal fat pad advancement [2].

Studies have shown that the facial artery can vary in anatomy, and the absence of a fully developed facial artery prevents the FAMM flap from being harvested [41]. Hatoko et al. discussed the use of the FAMM flap for repairing a defect in the mandibular vestibule [7]. The dissection begins at the far end of the flap by making a cut through both the mucosa and the buccinator muscle. Once the facial artery has been located, clamped, and divided, the flap is raised from the tissue layer directly underneath the facial vessels, while only lifting a small amount of the surrounding buccinator and orbicularis muscles near the oral commissure. Once lifted, the flap is then rotated downwards to cover the mandibular vestibule defect. After the flap is taken, the donor site is primarily closed while being careful not to disturb the Stensen's duct [7]. Some researchers have developed a technical variant of the FAMM flap, which involves isolating the flap on the facial vessels, bringing it to the neck, and then tunneling it on the lingual side of the mandible to repair lateral tongue defects [13]. Akali et al. presented a case report where the FAMM flap is used for reconstructing a partial pharyngeal defect following a total laryngectomy [13]. Using a sterile Doppler intraorally, the territory of the left buccal mucosa's facial artery is marked. A flap of 2 x 4 cm is marked, while keeping the opening of the Stensen's duct superiorly. After deepening the mucosal incision through the buccinator muscle, dissection continued, leading to the facial vessels. The facial vein and artery are ligated overhead the superior margin. After intraoral dissection alongside the facial pedicle, the intraoral flap area is connected to the cervical part. Finally, the islanded flap is tunneled medial to the marginal mandibular nerve entirely pedicled on the facial vessels [13]. The mucosal island variation of the FAMM flap may be a suitable option for reconstructing the nasal lining in certain situations. This variation also reduces tension on the flap and eliminates any risk of trapping the epithelium in the subcutaneous tunnel [39].

According to Pompei et al., after removing cancerous tissue, a flap is created of cheek mucosa that measured 8 x 3 cm and is oriented in the direction of squamous carcinoma. The flap is positioned at a 90-degree angle to the projection of the facial vessels, and then dissected in an anteroposterior direction and exposed the facial artery over a length of 3.5 cm above and below the flap entrance. After mobilizing the vascular pedicles and ligating the labial artery, they transposed the transverse (t)-FAMM flap superoposteriorly and sutured it to the remaining mucosa of the hard palate. They also harvested and transposed a contralateral t-FAMM flap. Finally, they reconstructed the entire soft palate by suturing the two flaps together [43]. To reconstruct the inside of the mouth, it is recommended by the authors to first make an incision through the mucosa and buccinator. Next, the facial artery at the base of the flap has to be identified and cut. Then, incisions are made to create the flap, including part of the deep layer of the orbicularis oris muscle. If there is enough soft tissue for adequate venous drainage, the surgeon may isolate the base of the flap. Finally, the donor defect is closed in two layers, muscle and mucosa, by being careful to avoid the opening of the Stenson's duct, or a split-thickness skin graft may be used to cover it [5]. Asairinachan et al. suggested that the use of a FAMM flap is recommended for reconstructing the lateral pharyngeal wall in certain patients undergoing transoral robotic surgery for oropharyngeal cancer. This technique provides the introduction of healthy and vascularized tissue to the resection area, aiding in healing and preventing complications that may arise from poor wound healing [45,46]. Using an anterosuperiorly based FAMM flap is a feasible choice for repairing large anterior hard palate defects. It is important to ensure sufficient venous drainage. This flap eliminates the need for multi-stage tongue flap repairs in patients with open maxillary arches [47]. A modified FAMM flap technique can be used to repair a defect on the side of the tongue after removal. The technique involves taking an island flap of buccinator muscle and mucosa, which is attached to the facial vessels. The flap is brought through the neck and into the oral cavity using a lingual tunnel. This modification increases the flap's rotation and eliminates the need for a second pedicle section. This technique was termed an "islanded FAMM flap" by Joseph et al. [48].

Joseph et al. described an islanded FAMM flap for tongue reconstruction [48]. The left lateral border of the tongue was surgically removed with an appropriate margin, and the resulting defect size was noted. Selective neck dissection was also carried out using a horizontal neck crease incision. A flap of healthy buccal mucosa was outlined and marked, with care taken to preserve the parotid duct opening. The flap was dissected carefully, identifying and ligating the facial artery and vein. The buccal fat pad was found while separating the buccinator and inferiorly, the flap was detached from the alveolar margins of the teeth. The facial vessels and marginal mandibular nerve were identified and preserved. The mobilized flap was pedicled on the facial vessels and tunneled lingually to reconstruct the lateral tongue defect while the buccal mucosa defect was covered with the harvested buccal fat pad [48]. Berania et al. have offered some tips and tricks for FAMM flap surgery. The flap design should follow the established landmarks, and if the flap width is reduced, the posterior incision should be moved anteriorly rather than placing the anterior incision posteriorly. Use needle tip cautery in the "cut" mode when performing mucosal incisions to minimize bleeding. To overcome mucosal contraction when using the flap for septal perforation, harvest the flap a few millimeters more than required. For anterior septum perforation, the gingivolabial incision is the shortest and safest approach. For posterior or skull base defects, other techniques such as the Caldwell-Luc approach may be used. If a reconstruction attempt with a FAMM flap fails, a second surgery may be performed using a contralateral FAMM flap, but identifying the initial cause of failure is necessary [49].

A buccal flap measuring up to 9 by 4 cm can be created and harvested using either anterograde or retrograde flow, with the focal point being the facial artery. When donor site defects measure 3 cm or less, the site is closed primarily in two layers. However, in cases of wider defects, primary closure of the donor site is not recommended to avoid trismus [1]. Massarelli et al. reported a case of reconstruction of a full-thickness cheek defect with a chimeric facial artery free flap [38]. The author explained that this method can offer an effective way to reconstruct medium-sized, full-thickness cheek defects by using tissue similar to the one lost, and with little to no harm to the donor site [38]. The versatility of the FAMM flap in reconstructing soft palatal defects after tumor removal or radiation is enhanced by islanding and tunneling modification, which provides a greater range of rotation and eliminates the need for a second-stage revision procedure [37].

**Figure 1:**
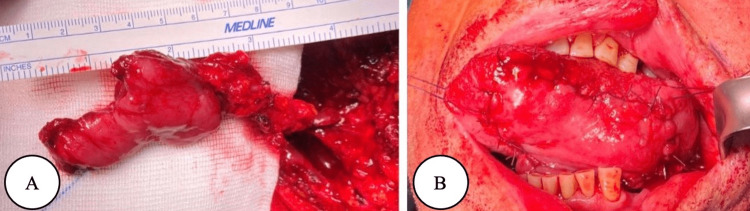
(A) The FAMM flap with a facial artery. (B) Tongue reconstruction with the FAMM flap Copyright: © 2021 by the authors. Licensee MDPI, Basel, Switzerland. This article is an open access article distributed under the terms and conditions of the Creative Commons Attribution (CC BY) license (https:// creativecommons.org/licenses/by/ 4.0/) [11]

Sohail et al. suggested choosing the FAMM flap as the primary method for closing large anterior palatal fistulas related to alveolar cleft, as it results in a shorter total operative time and fewer early postoperative complications [36]. The local chimeric flap based on the combination of tunnelized FAMMIF and submandibular gland flap provides additional options for reconstructing medium-sized defects in patients who are not ideal candidates for free flap reconstructions following oral cancer surgery [50]. If the flap is less than 3 cm wide, the donor site can be closed primarily. However, if excessive tension raises concerns over potential contracture, the donor site can choose to be skin grafted, left to granulate, or closed with buccal fat pad advancement [2]. Saboury et al. discussed a rare case of Rapp-Hodgkin syndrome; the authors further recommended the use of a combination of FAMM and inferior turbinate flaps for the treatment of the wide cleft [51]. As mentioned by Pribaz et al., the FAMM flap, which is a nearby flap, is suitable for reconstructing the lip and vermilion. While it is not exactly the same as the natural lip, it shares numerous similarities, which make it a highly favorable choice for lip reconstruction [52]. The use of the FAMM flap is demonstrated in Figure 1.

Survival rate

**Figure 2:**
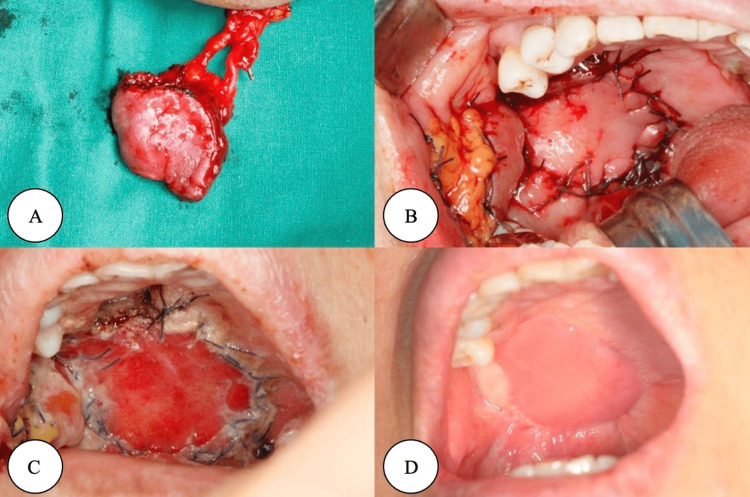
(A) Extraorally transposed t-FAMMIF. B) Closing the palatal defect with t-FAMMIF. C) Postoperative day 1. D) After 3 months The Author(s). 2017 Open Access. This article is distributed under the terms of the Creative Commons Attribution 4.0 International License (http://creativecommons.org/licenses/by/4.0/), which permits unrestricted use, distribution, and reproduction in any medium, provided you give appropriate credit to the original author(s) and the source, provide a link to the Creative Commons license, and indicate if changes were made [37]

The FAMM flap is a versatile and reliable option for head and neck reconstruction, particularly in oral cavity defects. Indications for the use of the FAMM flap include reconstruction of the floor of the mouth [8], alveolar cleft surgery [33], nasopharyngeal reconstruction [4], and tongue defect repair [29]. In addition, the FAMM flap has been applied in lower eyelid fornix deepening [28] and closure of large anterior palatal fistulas [36]. Contraindications for using the FAMM flap include a compromised or absent facial artery, as the flap relies on the blood supply provided by the facial artery [34]. Additionally, patients with a history of radiation therapy in the area may have compromised vasculature, which can pose a risk to flap survival [37]. Oncologic safety is another consideration, as the FAMM flap should not be used in cases where there is a risk of transferring malignant cells from the donor site to the recipient site [12]. The use of t-FAMMIF is demonstrated in Figure 2.

Complications

There are several minor complications that are commonly associated with the use of the FAMM flap in the literature. These include partial or complete flap necrosis, dehiscence, venous congestion, hematoma, and infections [2,51]. There are two factors that are highly related to an increased risk of having partial flap necrosis: previous neck dissection and previous radiation therapy [1]. Lahiri et al. reported two cases with partial necrosis and one with complete necrosis following venous congestion and stated that this complication can be prevented by widening the pedicle to maintain the flap vascular integrity [46]. Another cause of partial and complete necrosis is the failure to centralize the flap over the artery to ensure that the artery is included entirely in the flap [17,18].

According to Ashtiani et al., to prevent partial or complete necrosis, it is crucial to handle the flap without any twisting or stretching of the pedicle [18]. Pan et al. stated that increasing the flap size will increase the amount of dehiscence at the donor site [29]. Another factor that contributes to suture line dehiscence is suturing the flap to thin mucosa [17]. Ferarri et al. found that flap infection can be associated with suture dehiscence, which can resolve with daily dressing [12]. Venous congestion is another common complication, Joshi et al. reported that all 17 cases in their study resulted in venous congestion and mentioned that it resolved on its own after 24-48 hours by conservative management alone [5]. Benjamin et al. stated that it is expected to have venous congestion when the FAMM flap is used for the reconstruction of tongue defects [20]. Jowett et al. identified a complication arising from an injury to the orbicularis oris muscle body or its innervation, which resulted in the loss of perioral rhytids superior to the ipsilateral upper lip. Additionally, there is a risk of injuring the terminal branches of the infraorbital nerve due to their close proximity to the facial artery around the superior limit of an inferiorly based FAMM ﬂap, which can lead to upper lip anesthesia. To avoid these two possible injuries, it was suggested that the superior extension of the flap should be limited when a long flap is not necessary [43,44].

Trismus or limited mouth opening is another commonly reported complication [28], which was reported by the majority of the articles included in this review [3,5,15,24,26,27,28,29,42,46]. Some articles stated that the range of mouth opening returned to normal after varying periods ranging from 3 to 12 months after the patients engaged in mouth-opening exercises and massage [3,28]. Pan et al. suggested that it is better to keep the flap size smaller than 3.5 × 3.5 cm, in order to decrease the chance of postoperative trismus [29]. Intraoral hemorrhage can occur as a postoperative complication after using the FAMM flap due to the erosion of the fascial artery; this can be managed by immediate hemostasis [20].

There is a burden of sacrifice, which involves not including the facial vein in the flap to reduce the flap bulk, which increases the risk of venous congestion; this complication of either resulting in a bulky flap or venous congestion can be managed by utilizing the spacer FAMM flap modification in reconstructing oronasal fistula [21]. Mandibular nerve injury was reported by two articles, which resolved after three months [15,29]. Akali et al. reported mandibular nerve mild paresis caused by nerve traction that resolved after six months [13].

## Conclusions

Based on our findings, the use of the FAMM flap can lead to a significant improvement in facial defect reconstruction by ensuring a good blood supply from the facial artery. This review illustrates that the FAMM flap can be used for various facial reconstruction procedures. However, several points must be considered by the practicing surgeon. A compromised or absent facial artery, a history of radiation therapy, and oncologic risk are the main contraindication for using the FAMM flap. Moreover, the surgeon should have sufficient knowledge regarding the postoperative complications including necrosis, dehiscence, venous congestion, hematoma, and infections as well as the proper management of these complications. In addition, trismus or limited mouth opening was a commonly reported complication of this flap, which was reported by the majority of the articles included in this review.
